# A robust and accurate single-cell data trajectory inference method using ensemble pseudotime

**DOI:** 10.1186/s12859-023-05179-2

**Published:** 2023-02-20

**Authors:** Yifan Zhang, Duc Tran, Tin Nguyen, Sergiu M. Dascalu, Frederick C. Harris

**Affiliations:** Department of Computer Science and Engineering, University of Nevada, Reno, Reno, NV USA

**Keywords:** Single cell, Pathway, Trajectory inference, Pseudotime

## Abstract

**Background:**

The advance in single-cell RNA sequencing technology has enhanced the analysis of cell development by profiling heterogeneous cells in individual cell resolution. In recent years, many trajectory inference methods have been developed. They have focused on using the graph method to infer the trajectory using single-cell data, and then calculate the geodesic distance as the pseudotime. However, these methods are vulnerable to errors caused by the inferred trajectory. Therefore, the calculated pseudotime suffers from such errors.

**Results:**

We proposed a novel framework for trajectory inference called the **s**ingle-**c**ell data **T**rajectory inference method using **E**nsemble **P**seudotime inference (scTEP). scTEP utilizes multiple clustering results to infer robust pseudotime and then uses the pseudotime to fine-tune the learned trajectory. We evaluated the scTEP using 41 real scRNA-seq data sets, all of which had the ground truth development trajectory. We compared the scTEP with state-of-the-art methods using the aforementioned data sets. Experiments on real linear and non-linear data sets demonstrate that our scTEP performed superior on more data sets than any other method. The scTEP also achieved a higher average and lower variance on most metrics than other state-of-the-art methods. In terms of trajectory inference capacity, the scTEP outperforms those methods. In addition, the scTEP is more robust to the unavoidable errors resulting from clustering and dimension reduction.

**Conclusion:**

The scTEP demonstrates that utilizing multiple clustering results for the pseudotime inference procedure enhances its robustness. Furthermore, robust pseudotime strengthens the accuracy of trajectory inference, which is the most crucial component in the pipeline. scTEP is available at https://cran.r-project.org/package=scTEP.

## Background

Since the advent of single-cell RNA sequencing technology, researchers can study the dynamic cellular process at the resolution of an individual cell. Some dynamic processes such as cell differentiation, cell development, and cell fate decisions can be analyzed using the gene expression matrix and modeled by generating the graph showing the stage and pseudotime of cells. The development of single-cell RNA-sequencing technology has made enormous progress in scale, from analyzing dozens of cells to millions of cells. Therefore, the research in single-cell data is enhanced.

Many trajectory inference methods have been developed in recent years. We categorize the trajectory inference methods into two categories based on how they construct the trajectory. The **first category** of trajectory inference approaches is based on the minimum spanning tree (MST) algorithm, which attempts to infer the developmental trajectory of single-cell data. Monocle [1], The first pseudotime inference method, utilized the MST algorithm on individual cells to find the longest path and assign the pseudotime of each cell. Monocle2 [2] learns the cell trajectory using the MST algorithm and updates the cell positions by shifting cells toward the nearest vertex in the MST. Monocle2 then repeats this procedure until the cell trajectory and positions are stable. It finally calculates the pseudotime of the cells by their geodesic distance along the MST from the root vertex. Tools for Single Cell Analysis (TSCAN) [3] run the MST algorithm on clusters to construct a cluster-based MST, then orders the cells by orthogonally projecting cells onto the edges of the MST. It is worth mentioning that the total computation of the MST algorithm is reduced significantly by running on the cluster level instead of the cell level. Waterfall [4] is similar to TSCAN, it constructs the MST on clusters that are used as the trajectory, and calculates pseudotime by orthogonally projecting cells onto edges. Slingshot [5] constructs trajectory using the MST algorithm. When calculating pseudotime, Slingshot proposed a simultaneous principal curves algorithm to construct smooth curves from the MST, it then projects cells onto the smooth curves instead of MST edges.

The **second category** is the graph-based trajectory inference method. Diffusion pseudotime (DPT) [6] utilizes a weighted k-nearest-neighbor (KNN) algorithm to construct the trajectory of the cells. Then diffusion pseudotime algorithm is introduced to calculate the pseudotime of cells in what they call the ‘diffusion map space’. Partition-based graph abstraction (PAGA) [7] first compresses and denoises original data and constructs what they describe as a symmetrized KNN-like graph. It then finds the community of vertices using the Louvain [8] algorithm to partition this KNN-like graph. Monocle3 [9] generates the trajectory using the principal graph algorithm. It then calculates the geodesic distance of cells from the user-selected root node in the trajectory as the pseudotime. URD [10] uses a KNN graph between transcriptomes in gene expression space to construct trajectory. It then calculates the pseudotime of cells by utilizing the simulating diffusion algorithm to determine the distance of cells from the root.

The **third category** is the RNA velocity assisted trajectory inference method. VeTra [11] utilized RNA velocity vectors to construct multiple directed graphs that are obtained from lineage tracing to determine the transition state of cells based on *k* nearest neighbors of cells. Then, VeTra constructs independent cell transition paths by identifying weakly connected components. Lastly, those transition paths are clustered together to obtain trajectory. The pseudotime of cells is calculated by projecting them onto the principal curve which is obtained from lineages of trajectory. Cytopath [12] utilized RNA velocity to infer the root and terminal states. By combining the cell-to-cell transition probability matrix and cell states, Cytopath constructs multiple simulations of trajectories that are used to assign cell states. The pseudotime was estimated from those trajectories.

However, these existing methods have some drawbacks. Firstly, the existing methods have poor scalability in efficiency and accuracy. When the total number of cells reaches hundreds of thousands, the execution time increases rapidly and the accuracy drops. Furthermore, this situation becomes worse when the number of cells exceeds 10,000. Secondly, the existing methods do not utilize pathway information [13]. Pathway analysis is a very effective methodology to enhance the ability of gene expression analysis. It strengthens gene expression analysis by dividing genes related to each other into the same group. However, the existing trajectory inference methods did not utilize this effective tool. Thirdly, when conducting trajectory inference, users usually know that one cell or a group of cells is the starting point of the trajectory. Therefore, we can use the relative relationship between the remaining cells or clusters with the starting point (e.g. Euclidean distance) as additional information to construct the trajectory. However, many existing methods only use the starting point information to define the starting point of its generated trajectory. Fourthly, most developed trajectory inference methods in recent years use clustering to generate a graph that represents the trajectory at the cluster level, the pseudotime is then calculated from the graph. It is therefore very susceptible to errors in clustering and graph construction.

To solve these problems, we propose **s**ingle-**c**ell data **T**rajectory inference method using **E**nsemble **P**seudotime inference (scTEP), which consists of four major parts. The first part is pathway gene set intersection which utilizes the pathway information and generates latent for all pathways. The second part is scDHA [14] clustering and dimension reduction, which consists of a non-negative kernel autoencoder and a variational autoencoder. scDHA achieved outstanding performance on both latent representation and clustering tasks. We utilized scDHA as a part of the pipeline to enhance the capacity of trajectory inference. The third part is pseudotime inference from multiple clustering results that generate more robust pseudotime results. The fourth part is pseudotime fine-tuned trajectory inference, which utilizes the pseudotime inferred from the previous part and fine-tunes the constructed graph by sorting the vertex according to its average pseudotime. We conduct extensive experiments on real data sets and the results show that scTEP outperforms state-of-the-art methods in accuracy and robustness.

## Results and discussion

In this section, we show the scTEP’s experimental results using two collections of data sets. First, we use the gold standard data sets collected in [15] to compare the performance of the trajectory inference methods. The gold standard collection consists of data sets where their ground truth trajectory is not obtained from the gene expression data. The gold standard collection had data sets of many trajectory types, such as linear, bifurcation, multi-bifurcation, tree, connected, and disconnected. We utilized 26 members of the gold data collection out of 27 (all of the Homo sapiens and Mus musculus data sets), of which 17 are linear data sets and the remaining 9 data sets are non-linear. Second, we demonstrate the pseudotime obtained by scTEP on larger linear data sets. We used six state-of-the-art methods shown to be most accurate in [15] and evaluated the pseudotime inference capacity of all methods using 1 example dataset of size 128 and 14 linear data sets ranging in size from 1,907 to 182,174 cells. It is worth noting that we assembled this collection to evaluate the performance across this spectrum of sizes.

### Gold standard data sets

To benchmark scTEP on branching data sets, we utilized all the Homo sapiens and Mus musculus members of the gold standard data sets collected by Saelens in [15]. This selected subset of the gold standard data sets consists of 17 linear data sets and 9 non-linear data sets. We conducted experiments on gold standard data sets to evaluate the trajectory inference capability of scTEP. In addition to their paper [15], Saelens et al. also developed a collection of R packages called dynverse to help researchers working on the trajectory inference task. The dynverse collection consists of four main packages, dynwrap [16], dynplot [17], dyneval [18], and dynmethods [19]. The dynmethods package contains state-of-the-art trajectory inference methods. The dynplot and dyneval packages provide the functionality to visualize and evaluate the output of dynmethods. The dynwrap package is committed to allowing the user to wrap their developed method in the formatting consistent with dynverse. Therefore, we can use dynplot, dyneval, and dynmethods to compare a user’s developed method with state-of-the-art methods.

To utilize the convenience of dynverse packages, we wrapped scTEP using the dynwrap, then used dyneval to conduct extensive experiments on the linear and non-linear gold standard data sets. Then, we compared scTEP with state-of-the-art methods. The dynwrap package used in the experiment is version 1.2.2. We selected 8 state-of-the-art methods that performed the best according to the accuracy results in [15] from the dynmethods package version 1.0.5., note that we wrapped the Monocle3 method using dynwrap since dynmethods did not contain it. Finally, we used dynplot and version 0.9.9 dyneval packages to visualize and evaluate the comparison.

**Fig. 1:**
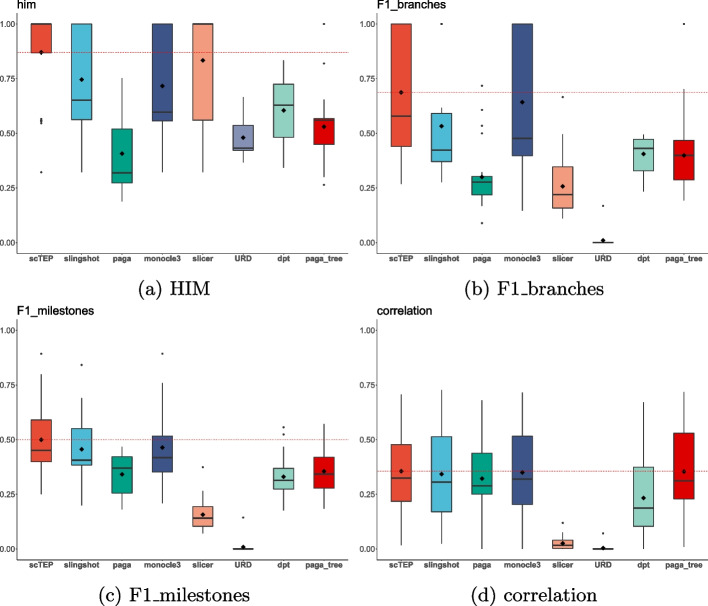
Box plots for HIM, F1 branches, F1 milestones, and correlation values for 26 gold standard data sets. The diamond shape in the box indicates the mean value of a method. The mean value of scTEP is also shown as a red dashed horizontal line for comparison. The scTEP outperforms other state-of-the-art trajectory inference methods by having the best mean values regarding all four metrics

To evaluate the performance of trajectory inference methods, we utilized the HIM (Hamming-Ipsen-Mikhailov distance), F1 branches, F1 Milestones, and correlation metrics in the dyneval package, and the experiment results using gold standard data sets are present in Fig. 1. Those metrics are also used in the [15] to evaluate the accuracy of 45 trajectory inference methods that generate different formatting outputs. To compare multiple methods, the dyneval package abstracts a method’s output trajectory into a uniform format that uses a milestone network and the assignment of cells within the milestone network to represent the trajectory and pseudotime of a method’s output. The HIM metric uses the adjacency matrix, with the length of edges within the milestone network as the values of its elements, to calculate the similarity in topology between two graphs regardless of the assignment of cells. F1 branches and F1 Milestones aim to compare the accuracy of cell assignment within the milestone network. To calculate F1 branches and F1 Milestones, the dyneval package first map cells to their closest branch and milestone, respectively, then use the F1 score to evaluate the accuracy of the cell assignment. The correlation metric represents the correlation between a method’s output cell geodesic distance from the starting point within the milestone network and ground truth. Saelens et al. [15] provide detailed descriptions and calculations of those metrics in the supplementary file.

The HIM metric indicates the similarity between the inferred trajectory by methods and the ground truth trajectory. In Fig. 1a, we present the box plot of HIM values of gold standard data sets. The average HIM of scTEP is 0.87. Slicer is the second-best method that achieved an average HIM of 0.83, which is shown in Fig. 1a as the diamond shape in the box and red dashed horizontal line. Note that although Slicer can infer nonlinear trajectories. However, all Slicer’s outputs are linear after being wrapped into milestone networks by dynwrap. Monocle3 and Slingshot are the third and fourth best methods and have an average of 0.74 and 0.71, respectively. The remaining methods performed significantly worse as shown in Fig. 1a. As for the accuracy of pseudotime inference, scTEP performed the best on both F1 branches (Fig. 1b) and F1 milestones (Fig. 1c). For F1 branches, scTEP’s average is 0.687. Monocle3 is the second-best method in terms of this metric, with an average of 0.642. Slingshot has a lower average of 0.53. As for the rest, they performed significantly worse. For F1 milestones, scTEP’s average is 0.5. The performance of Monocl3e and Slingshot is similar. The average F1 milestones of Monocle3 is 0.464. The Slingshot is a bit worse, with an average of 0.456. Lastly, Fig. 1d shows the correlation values. The scTEP has the best result of an average of 0.355. paga_tree is the second best, its average correlation is 0.354. Monocle3 and Slingshot are the third and fourth, with an average of 0.3498 and 0.342, respectively. Those methods all performed well in terms of correlation metrics.

Overall, we conclude that scTEP performed the best on gold standard data sets. It has better accuracy in both trajectory and pseudotime inference.

**Fig. 2:**
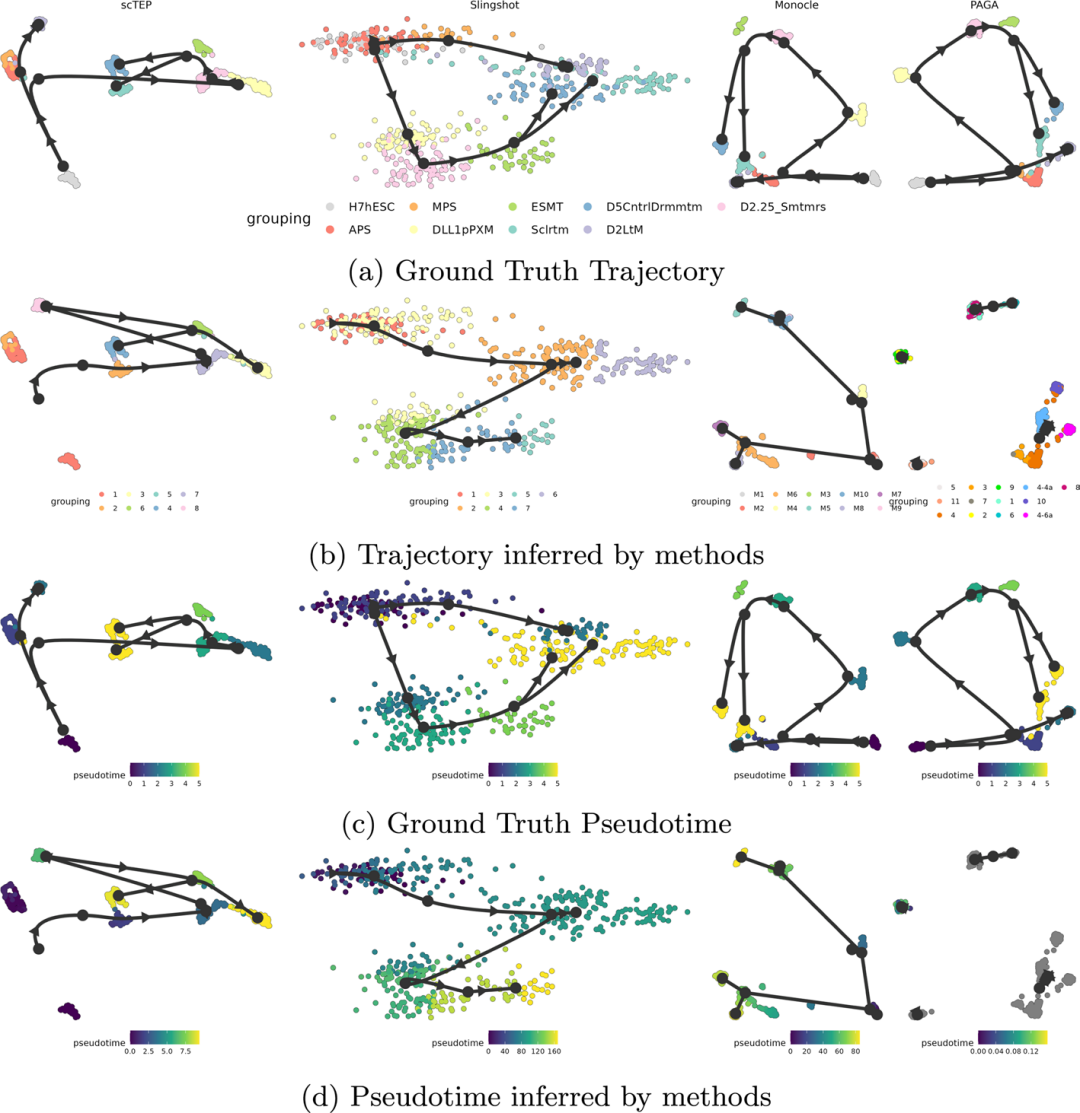
The visualization of ground truth, inferred trajectories, and pseudotime on the Mesoderm development loh data set. The landscape in the reduced dimension space provided by each method is colored by: **a** ground truth development stages, **b** trajectory inference results, **c** ground truth pseudotime, **d** pseudotime inferred by methods

Figure 2 shows the visualization of ground truth and trajectory inference results of scTEP and other three state-of-the-art methods on the Mesoderm development loh data set [20]. The NKT differentiation engel data set consists of nine types of cells: H7hESC, APS, MPS, DLL1pPXM, D2.25_Smtmrs, ESMT, D5CntrlDrmmtm, Sclrtm, D2LtM. Figure 2a shows the ground truth trajectory and cell types of the Mesoderm development loh data set in reduced dimensional spaces output by scTEP, Slingshot, Monocle3, and PAGA, respectively. The solid black dots in the figure show the center of cell types, which is the ground truth cell types of the data set in Fig. 2a and the cell type identified by trajectory inference methods in Fig. 2b. The solid black lines with arrows connecting the dots indicate the development trajectory of cells. The trajectory of the Mesoderm development loh data set is a tree. The H7hESC is the root cell type, all the other types of cells are derived from H7hESC. Then divided into two branches starting with APS and MPS. The MPS branch is linear and followed by D2LtM cells. The APS branch is followed by DLL1pPXM, D2.25_Smtmrs, and ESMT cells. Then bifurcate after ESMT cells into D5CntrlDrmmtm and Sclrtm cells. Figure 2b shows the inferred trajectory by scTEP, Slingshot, Monocle3, and PAGA. scTEP clustered cells into 8 groups. It missed the branch consisting of MPS and D2LtM. It correctly identified the second differentiation point near the end of another branch. Slingshot identified the branch consisting of MPS and D2LtM. However, it failed on another branch by connecting another branch after the D2LtM cells. It also connects D5CntrlDrmmtm cells after the branch of MPS and D2LtM cells. Monocle3 identified a branch consisting of MPS and Sclrtm cells, then ended with differentiated into D5CntrlDrmmtm and D2LtM cells. Another branch has four start cell types, which is far from the ground truth shown in Fig. 2a. PAGA generated four independent trajectories. It also generated cycles in group 4 which doesn’t exist in the ground truth trajectory. Overall, PAGA’s output is significantly worse than the rest methods.

Figure 2c shows the ground truth pseudotime of Mesoderm development loh data set. It is worth mentioning that the D2LtM and D3GARPpCrdcM cells has a smaller pseudotime than cells on another branch. Figure 2d shows the pseudotime inference results of scTEP, Slingshot, Monocle3, and PAGA. For scTEP, the pseudotime for D5CntrlDrmmtm and Sclrtm cells is incorrect, the rest cells are close to the ground truth. For Slingshot, the pseudotime of ESMT, D5CntrlDrmmtm, and Sclrtm cells are incorrect. For Monocle3, the pseudotime of MPS, ESMT, D5CntrlDrmmtm, and Sclrtm cells are incorrect. Since PAGA generated four independent trajectories, it failed on calculating the cells are not connected to the starting point. Therefore, most cells don’t have pseudotime.

### Our collection

We compared scTEP with six methods that are recognized as the best on linear data sets according to [15]. These methods are also widely used to solve the pseudotime inference problem. Note that TSCAN and SCORPIUS are only able to generate linear output. We collected the data sets presented in Table 1, then converted the raw data into the SinglecellExperiment object for the convenience of comparison. Table 1 summarizes the characteristics of linear data sets in our collection. These linear data sets are labeled with developmental stages, such as known cell types or the time point of the cell, and these labels were used to evaluate the accuracy of the various methods.

**Table 1:** Description of the linear single-cell data sets

Data set	Tissue	Size	Class	Accession ID
1. Goolam	Mouse Embryo	124	5	E-MTAB-3321 [21]
2. Manno (Mouse)	Mouse Brain	1,907	6	GSE76381 [22]
3. Han	Mouse Embryo	3,105	3	GSE108097 [23]
4. Manno (Human)	Human Brain	4,029	12	GSE76381 [22]
5. Yuzwa	Mouse Embryo	6,316	4	GSE107122 [24]
6. Pijuan	Mouse Embryo	16,936	2	E-MTAB-7324 [25]
7. Green	Mouse Testis	22,954	3	GSE112393 [26]
8. Hochgerner	Mouse Embryo	24,185	8	GSE104323 [27]
9. Vladoiu	Mouse Brain	55,325	9	GSE118068 [28]
10. Weinreb (Cytokine)	Mouse Blood	65,076	4	GSE140802 [29]
11. Ernst	Mouse testis	84,018	11	E-MTAB-6946 [30]
12. Delile	Mouse Embryos	97,771	5	E-MTAB-7320 [31]
13. Park	Human Thymus	129,493	3	E-CURD-79 [32]
14. Weinreb (inVitro)	Mouse Blood	130,887	3	GSE140802 [29]
15. Weinreb (inVivo)	Mouse Blood	182,174	3	GSE140802 [29]

In our experiments, we used the following packages: (i) TSCAN version 1.24.0 from Github [33], (ii) SCORPIUS version 1.0.7 from CRAN, (iii) Slingshot version 1.4.0 from Bioconductor, (iv) Monocle3 version 1.0.0 from Bioconductor, (v) PAGA through Scanpy version 1.7.2, (vi) VIA [34] through pyVIA version 0.1.7. TSCAN and SCORPIUS are designed to work without prior information of start or end cells. The start cell type prior information is provided to the rest methods in the experiments.

Since these data sets were labeled with developmental stages, we used the correlation between inferred pseudotime and ground truth developmental stages as a criterion to evaluate the pseudotime inference accuracy of these methods. Table 2 summarizes the evaluation results of scTEP and 5 state-of-the-art methods on 15 linear data sets presented in Table 1, as well as the mean, median, and variance of correlation values.

The average and median correlation values of scTEP are 0.61 and 0.66, respectively. scTEP is the highest among all compared methods. The second-best method, Monocle3 achieved an average correlation of 0.53. Slingshot has a slightly lower average correlation of 0.50. PAGA and VIA achieved a significantly lower average correlation of 0.32 and 0.30, respectively. The remaining methods, TSCAN and SCORPIUS have an average of around 0. We deduced that due to the absence of the ability to utilize the start cells prior information, they failed to identify meaningful pseudotime.

**Table 2:** The trajectory inference results on 15 data sets

data set	scTEP	Slingshot	TSCAN	SCORPIUS	PAGA	Monocle3	VIA
1. Goolam	0.89	0.90	$-0.27$	$-0.28$	0.29	0.86	$-0.63$
2. Manno (Mouse)	0.27	0.30	$-0.34$	0.17	0.33	0.46	$-0.18$
3. Han	0.73	0.69	$-0.76$	0.39	0.66	0.19	0.27
4. Manno (Human)	0.68	0.77	$-0.76$	0.81	0.41	0.42	0.17
5. Yuzwa	0.66	0.41	0.62	$-0.66$	0.66	0.35	0.62
6. Pijuan	0.62	0.58	0.20	$-0.82$	0.56	0.67	0.86
7. Green	0.72	0.75	$-0.50$	$-0.23$	0.08	0.84	0.28
8. Hochgerner	0.67	0.57	0.23	$-0.39$	0.07	0.76	0.72
9. Vladoiu	0.50	0.16	0.31	0.13	0.23	0.59	0.73
10. Weinreb (Cytokine)	0.50	0.21	$-0.32$	0.05	0.30	0.40	NA
11. Ernst	0.68	0.73	0.65	-0.36	0.09	0.80	0.49
12. Delile	0.71	0.49	$-0.50$	-0.66	NA	0.35	0.31
13. Park	0.33	0.20	$-0.58$	0.48	0.06	0.70	$-0.08$
14. Weinreb (inVitro)	0.53	0.41	0.09	0.58	0.43	0.20	NA
15. Weinreb (inVivo)	0.64	0.36	$-0.09$	0.33	0.34	0.43	NA
Mean	**0.61**	0.50	$-0.13$	$-0.03$	0.32	0.53	0.30
Median	**0.66**	0.49	$-0.27$	0.05	0.315	0.59	0.30
Variance	**0.025**	0.054	0.216	0.248	0.047	0.051	0.19

**Fig. 3:**
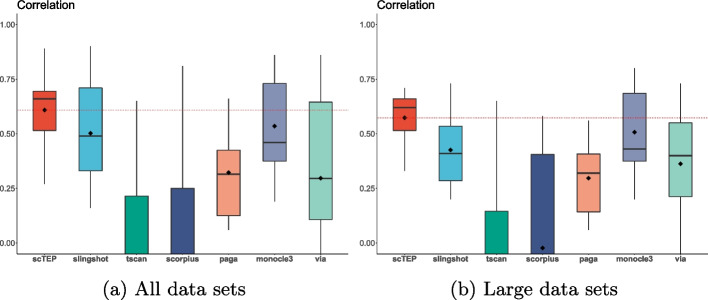
Box plots for correlation values for 15 real scRNA-seq data sets. The diamond shape in the box indicates the mean value of a method. The mean value of scTEP is also shown as a red dashed horizontal line for comparison. The scTEP outperforms other state-of-the-art trajectory inference methods by having the best average correlation value. **a** The correlation values of all data sets. **b** The correlation values of data sets that are larger than 50,000 cells

Figure 3a shows the box plot of pseudotime inference results of scTEP and the 6 state-of-the-art methods on all 15 linear data sets. We conclude that scTEP outperformed the other method significantly by having the highest mean and median correlation values. As for variance, scTEP also is significantly better than the compared methods. We conclude that scTEP has better robustness. For the rest methods, Slingshot and Monocle3 are working promisingly, while PAGA and VIA lag behind in accuracy. Both TSCAN and SCORPIUS have an average correlation of around 0 and fail on many data sets. Overall, scTEP enhanced the pseudotime inference ability over state-of-the-art methods.

In recent years, the cell size of single-cell RNA sequence data sets has reached more than a million, a significant increase compared to several years ago. The increasing number of cells makes trajectory inference harder. Cell clustering is a fundamental component in the trajectory inference pipeline and is more challenging to conduct on a large data set. The error in clustering affects trajectory inference in two aspects. First, the wrong number of clusters will cause errors in the graph that is the basis of the cell development trajectory. For instance, the graph construction method can generate extra branches that don’t exist in the ground truth trajectory because of incorrect clusters from clustering results. Second, most state-of-the-art methods build the graph at the cluster level instead of the individual cell level. There are always a certain amount of cells grouped into incorrect clusters because of the intrinsic property of the clustering task. Therefore, the errors from the clustering procedure will result in projecting those cells to the wrong position in the graph. Hence, the inferred pseudotime for those cells is incorrect. Increasing cell size also affects the dimension reduction component in the trajectory inference pipeline. It is much harder to generate a low-dimensional space that makes the same group of cells closer and cells from different groups farther. We observed that the landscape in the low-dimension space becomes dense when the data set size is beyond several thousand. In the circumstance of multiple groups of cells overlapping with each other in the low-dimensional space, the trajectory inference task becomes much more challenging.

When the data set size is greater than 50,000 cells (data sets 9–15 of Table 2), scTEP also achieved the best accuracy in terms of correlation, an average of 0.55. Monocle3 performed second with an average of 0.495. The rest of the comparison methods suffer from the large data set size, and the performance degrades significantly. Slingshot is the third-best with an average of 0.365. Figure 3b shows the box plot of 7 data sets with more than 50,000 cells. The performance of scTEP, Slingshot, PAGA, and Monocle3 dropped from their average over all 15 data sets. The experiment validates that trajectory inference is a more challenging task for large data sets, and scTEP performed the best among the 5 state-of-the-art methods compared.

In addition to the overall best accuracy, scTEP achieved better robustness over all the data sets. Scrutinizing the data sets individually, we observed that all the comparison methods performed well on most data sets while failing on a few data sets. Although Monocle3 performed better than Slingshot on large data sets, the overall performance of Slingshot and Monocle3 is promising according to the results exhibited in Table 2 and Fig. 3. Although both Slingshot and Monocle3 achieved overall good results, Slingshot had an abnormally low accuracy on the four data sets of Vladoiu, Weinreb (Cytokine), and Park. Monocle3 suffers the same issue on Han and Weinreb (inVitro) data sets. PAGA failed on Green, Hochgerner, Vladoiu, Ernst, Delile, and Park data sets. Especially its correlation with ground truth on Green, Hochgerner, Ernst, and Park are 0.08, 0.07, 0.09, and 0.06, respectively. That is barely better than a random guess. In particular, PAGA doesn’t work on the Delile data set. Therefore, scTEP has better accuracy and robustness on large data sets.

**Fig. 4:**
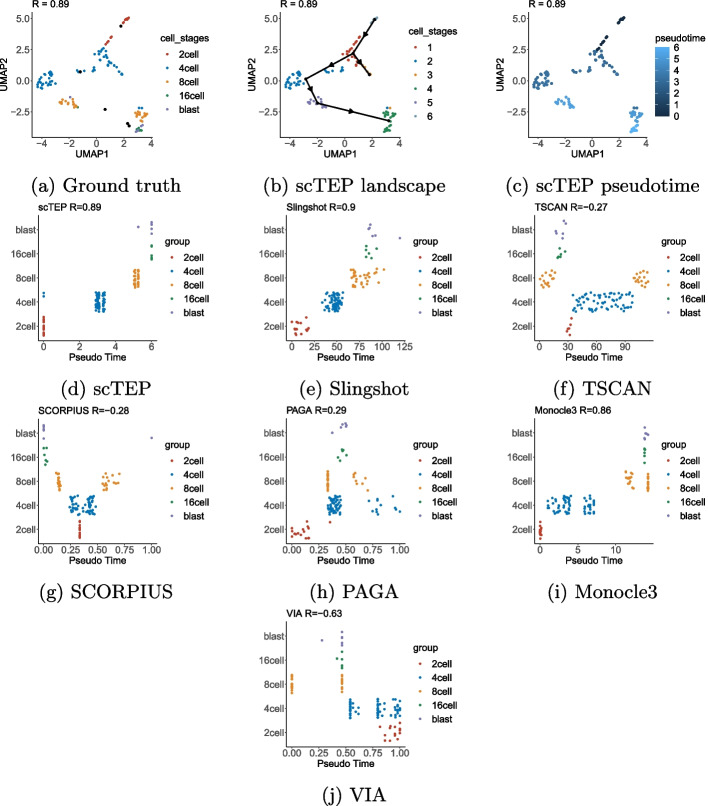
Visualization and comparison on the Goolam [21] data set: **a** The landscape of Goolam data set using UMAP colored by ground truth development stages. **b** UMAP landscape colored by clustering results. **c** UMAP landscape colored by pseudotime. **d** The pseudotime of scTEP against ground truth development stages. **e** Slingshot. **f** TSCAN. **g** SCORPIUS. **e** PAGA. **i** Monocle3. **j** VIA

**Fig. 5:**
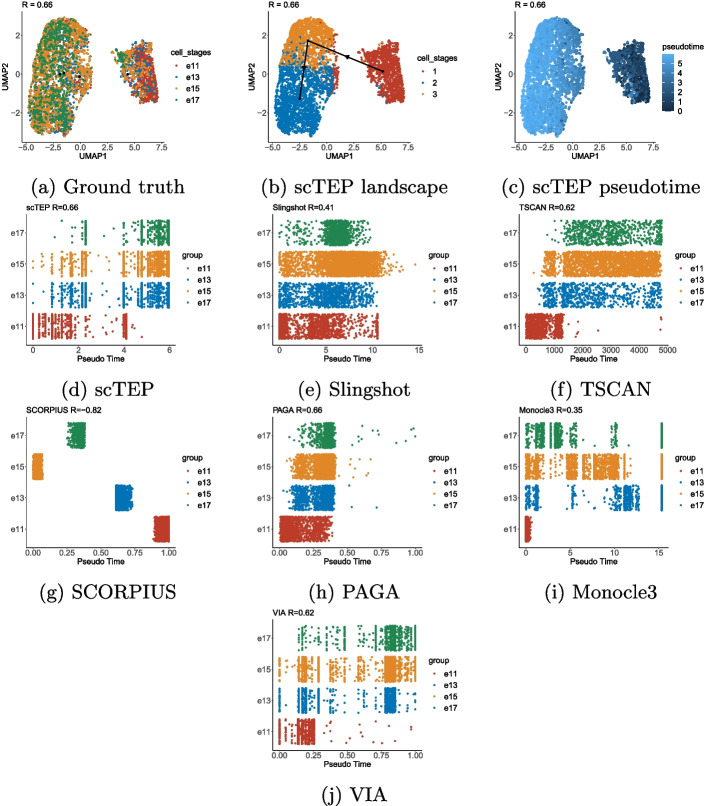
Visualization and comparison on the Yuzwa [24] data set: **a** The landscape of MouseCortex data set using UMAP colored by ground truth development stages. **b** UMAP landscape colored by clustering results. **c** UMAP landscape colored by pseudotime. **d** The pseudotime of scTEP against ground truth development stages. **e** Slingshot. **f** TSCAN. **g** SCORPIUS. **h** PAGA. **i** Monocle3. **j** VIA

Figure 4a–c show the landscape, trajectory, and pseudotime inferred by scTEP on the Goolam [21] data set in the two-dimensional space visualized by UMAP [35]. The Goolam data set consists of five cell types: 2*cell*, 4*cell*, 8*cell*, 16*cell*, and *blast*. The one imperfection in the scTEP’s output is that scTEP clustered 4*cell* into 3 groups and generated one additional lineage by mistake. However, the 3 4*cell* groups still have a very close pseudotime inferred, between 2*cell* and 8*cell* as shown in Fig. 4d. scTEP achieved a correlation of 0.89, the second among the compared methods. Figure 4c shows the landscape of the Goolam dataset colored by scTEP’s output pseudotime. The overall trend of the scTEP’s output pseudotime is consistent with the ground truth. Figure 4d–j shows pseudotime against development stages. scTEP has an almost perfect pseudotime except for 16*cell* and *blast* cells are close to each other. Slingshot correctly inferred pseudotime for 2*cell*, 4*cell*, and 8*cell*, and also failed with 16*cell* and *blast*. Some 8*cell* has a higher pseudotime than 16*cell* and *blast*. The outputs of TSCAN and SCORPIUS are overall incorrect. Both methods made 2*cell*, 4*cell*, and 8*cell* have a higher pseudotime than 16*cell* and *blast*, which is inconsistent with the ground truth. PAGA failed on 4*cell* and 8*cell* by assigning those cells a higher pseudotime than *blast*. Monocle3 has a similar output with Slingshot, 8*cell* cells are separated into two groups, but the inferred pseudotime for one group is higher than that for 16*cell* and *blast*. VIA’s inferred reversed pseudotime and 8*cell*, 16*cell*, and *blast* are intervened (Fig. 5a–c).

show the visualization of the Yuzwa data set and the trajectory inference results of scTEP methods. The Yuzwa dataset consists of four types of cells collected from four timepoints, namely *E*11, *E*13, *E*15, and *E*17. Figure 5a shows the landscape of the Yuzwa data set. We observed that four types of cells are not separable. Cells from multiple time points are located in two areas and overlap with each other. Although such a landscape is challenging for pseudotime inference, scTEP generated the correct linear trajectory and a correlation of 0.66, as shown in Fig. 5b. Overall, Fig. 5c shows the pseudotime pattern on the landscape is that the pseudotime of cells is increasing from right to left, which is consistent with the ground truth. The lower row of Fig. 5d–j shows the pseudotime against the development stages of scTEP and compared methods. scTEP has similar results with the landscape, the pseudotime for four types of cells are intervened. However, there is still a trend from *E*11 to *E*17. All methods output a pseudotime that intervened four cell types except for SCORPIUS. However, SCORPIUS output a pseudotime in reversed order. We conclude that scTEP consistently infers promising pseudotime when compared to state-of-the-art methods.

## Methods

**Fig. 6:**
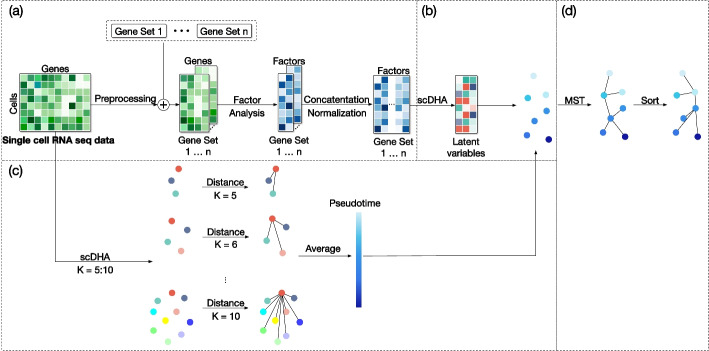
The architecture of our proposed **s**ingle-**c**ell data **T**rajectory inference method using **E**nsemble **P**seudotime inference (scTEP). It consists of four parts: **a** Data pre-processing and Pathway gene sets intersection, **b** scDHA [14] clustering and dimension reduction, **c** Ensemble pseudotime inference, and **d** Trajectory construction using MST algorithm on clusters and fine-tuned by Pseudotime

In this section, we first introduce the overall structure of the proposed pipeline and then discuss the details of the pipeline parts. Figure 6 shows the overall workflow of the pipeline consisting of four parts: (a) data pre-processing and pathway gene sets intersection, (b) scDHA clustering and dimension reduction, (c) ensemble pseudotime inference, and (d) trajectory inference.

### Software package and setting

In scTEP, we utilized the following packages: (i) org.Hs.eg.db [36] version 3.10.0 from Bioconductor, (ii) org.Mm.eg.db [37] version 3.10.0 from Bioconductor, (iii) Seurat [38] version 3.2.0 from CRAN, (iv) scDHA [14] version 1.1.2 from CRAN, (v) igraph [39] version 1.2.11 from CRAN, (vi) psych [40] version 2.1.6 from CRAN, (vii) doParallel [41] version 1.0.16 from CRAN. scDHA provides the dimension reduction and clustering functionalities, and igraph provides the functionality to construct the MST. Therefore, the scDHA and igraph packages have a greater influence on the results than the others.

### Data pre-processing

Figure 6a shows the data pre-processing procedure. The input for scTEP is single-cell RNA sequence data which comes in as an $m*n$ matrix representing the expression of *n* genes on *m* cells. There are several techniques used to normalize the single-cell data sets, such as raw counts, counts per million mapped reads (CPM), reads per kilobase million (RPKM), and transcript per million (TPM). One drawback of these normalization techniques is that some genes could have a much larger scale than others and become dominant when compared to other genes. To make the most of the gene expression profile, we first perform the log transformation (base 2) to rescale the raw expression count until the range of gene expression is smaller than 100. Another drawback of the gene expression matrix is that many genes collected don’t have a count read on any cell or only in a small portion of the cells. Since these columns are mostly 0, their contribution approaches 0 and wastes computation time. Therefore, those genes only expressed in very few cells should be removed from further analysis. We perform gene quality control by removing genes expressed in less than 20% of cells from the input.

### Pathway gene sets intersection

Figure 6a also shows the pathway gene sets intersection. There are tens of thousands of genes collected in an expression matrix. In a biological process, hundreds of thousands of genes work together corporately to direct the behavior of a cell instead of working alone. However, the relationships between genes during the development process are neglected in the previous methods. Those methods handle all genes indiscriminately and independently in dimension reduction and clustering tasks, Instead of utilizing the dependencies of genes. We believe that only a part of the genes contributes to the process of cell development. Therefore, we introduce the KEGG database and utilize it with an intersection operation with gene sets in the KEGG database to better learn the information about gene expression. The KEGG database collects and categorizes genes whose expression is related to each other. For instance, Homo sapiens (human) consists of 330 pathways, and the size of the individual pathway ranges from dozens of genes to fifteen thousand genes. We first select the corresponding pathway gene sets of the data set from KEGG, then intersect the genes in the expression matrix with each pathway to have an intersect gene expression matrix for all pathways. However, we expect to have some pathways that only have several genes matched in the gene expression matrix of the data set. We remove those pathways from the following computation. Heuristically, we set 10 genes as a threshold for pathway removal. We then have a gene expression submatrix for each gene set in the pathway. However, the intersected gene expression submatrix between pathways is on a different scale ranging from dozens to thousands of genes. Therefore, some pathways with significantly large sizes will be dominant if we analyze the intersected gene expression submatrix. We instead generate a latent representation for the individual pathway from the gene expression submatrix.

To learn the latent from pathways, we used the factor analysis function from the psych package to conduct factor analysis on all pathways’ gene expression matrix from the intersection and generate pathway factors. The output factor of each pathway will only be two dimensions, the factor analysis step further reduces the dimension of the gene expression matrix and meanwhile keeps maintaining information. Then, we concatenate the factors from pathways into one whole matrix, in which the dimension will be two times the number of pathways left. Note that we scrutinized the distribution of factor analysis results and observed that most of the values are between $-5$ and 5 with very few outliers outside of this range. Therefore, we apply the outlier cutting technique to set all the outliers to $-5$ or 5 based on their value. By applying intersection and factor analysis to the gene expression matrix, we significantly reduce the dimensions of the gene expression matrix, for example, from a total gene count of more than 20,000 to a few hundred. Therefore, the amount of computation of the following pipeline is reduced significantly.

**Fig. 7:**
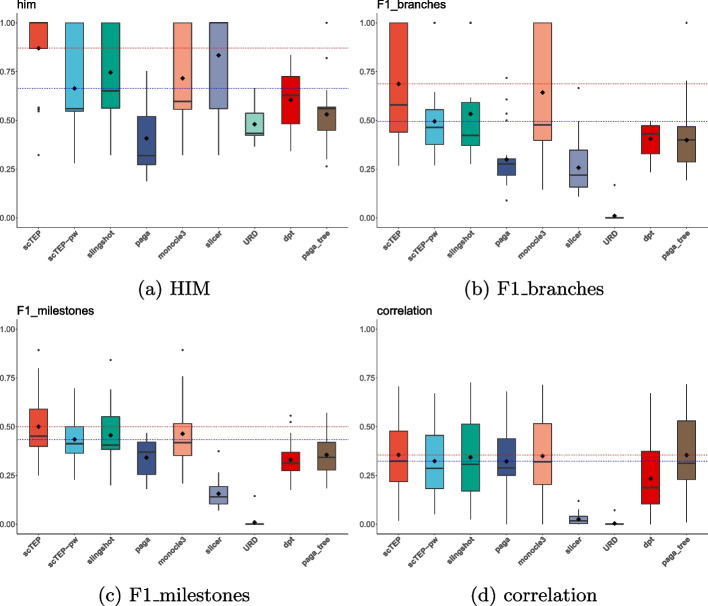
Box plots for HIM, F1 branches, F1 milestones, and correlation values for 26 gold standard datasets. The diamond shape in the box indicates the mean value of a method. The mean values of scTEP and scTEP without pathway gene sets intersection procedure (scTEP-pw) are also shown as a red and blue dashed horizontal line for comparison, respectively. The scTEP’s performance is degraded by removing the pathway gene sets intersection procedure

Given that the pathway gene sets intersection is one of the main components and contributions of scTEP, we experimented to validate its effectiveness. We tested scTEP without pathway gene sets intersection procedure (scTEP-pw) and evaluated its performance. Figure 7 shows the results of scTEP without pathway gene sets intersection procedure compared with other methods using gold standard datasets. Figure 7a and b show that scTEP-pw’s performance drops significantly in terms of HIM and F1 branches, respectively. On the other hand, scTEP-pw’s performance dropped less in terms of F1 milestones and correlation from the original scTEP, as shown in Fig. 7c and d. Overall, by removing the pathway gene sets intersection procedure, scTEP’s average value dropped 0.20, 0.19, 0.06, and 0.03 in terms of HIM, F1 branches, F1 milestones, and correlation, respectively. Those results demonstrated that the pathway gene sets intersection procedure is essential to scTEP. It is worth mentioning that HIM, F1 branches, and F1 milestones are metrics affected by the topology of the trajectory. Those are affected more significantly than the correlation of pseudotime.

**Fig. 8:**
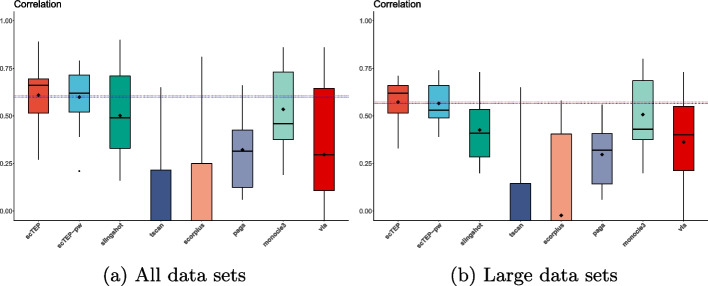
Box plots for correlation values for 15 real world datasets. The diamond shape in the box indicates the mean value of a method. The mean values of scTEP and scTEP without pathway gene sets intersection procedure (scTEP-pw) are also shown as a red and blue dashed horizontal line for comparison, respectively. The scTEP without pathway gene sets intersection procedure only degraded a trivial amount regarding the correlation. **a** The correlation values of all data sets. **b** The correlation values of data sets that are larger than 50,000 cells

Figure 8a shows the results of scTEP-pw compared with other methods using our collection datasets. The performance drop in terms of correlation is 0.01. Figure 8b shows the results of scTEP-pw compared with other methods using our collection datasets that are larger than 50,000 cells. scTEP-pw’s average correlation dropped from the original scTEP with only 0.0007. We speculate that the difference in performance is because the pathway gene sets intersection procedure is mainly influential for the generation of low-dimensional spaces. The calculation of correlation results shown in Fig. 8 used the pseudotime inferred from ensemble clustering results. It is more robust to variations in low-dimensional space. On the other hand, the dynverse package calculates all four HIM, F1 branches, F1 milestones, and correlation using the graph constructed in the low-dimensional space. Hence, those results are more vulnerable to the latent representation generated without the pathway gene sets intersection procedure.

### scDHA clustering and dimension reduction

Figure 6b shows the scDHA clustering and dimension reduction procedure. An Autoencoder is a type of neural network which aims to infer the output which contains the essential information from the input. Tran et al. proposed an encoder-decoder architecture generative deep neural network named scDHA [14]. scDHA consists of two core modules. The non-negative kernel autoencoder is the first module used to filter out insignificant genes or components and generate intermediate states. The stacked bayesian autoencoder based on a variational autoencoder(VAE) [42] is utilized as the decoder to project the high-dimensional intermediate states into low-dimension space, also known as latent. scDHA has demonstrated superior performance in single-cell data analysis, such as dimension reduction and clustering.

Considering that both dimension reduction and clustering are two fundamental steps in the trajectory inference pipeline, we choose to integrate scDHA into our proposed pipeline to conduct dimension reduction and clustering from learned factors of pathways. To demonstrate the importance of dimension reduction and clustering procedures, we tested replacing scDHA with three other dimension reduction algorithms (PCA, TSNE [43], and UMAP [35]) and four other clustering algorithms (K-means, Louvain [8], Leiden [44], and scCAN [45]) in scTEP. However, replacing scDHA with either of those algorithms will degrade the performance of scTEP.

In the proposed framework, we utilized scDHA to achieve two goals. The first goal is to apply scDHA six times with the parameter k (cluster number) set from 5 to 10 that runs clustering all the cells into k clusters, as shown in Fig. 6c. Then scTEP utilizes these six clustering results to produce a robust ensemble pseudotime for cells. The second goal is to generate the latent and clustering result with the automatically detected cluster number from intersected factors, as shown in Fig. 6b. The scTEP then utilized scDHA’s latent and cell clustering to learn a graph as the trajectory produced by scTEP.

### Ensemble pseudotime inference

Figure 6c shows the ensemble pseudotime inference procedure. The pseudotime inference task is crucial to trajectory inference. Most of the methods inferred the trajectory first and then use it to infer the pseudotime. For instance, the slingshot method constructs an MST and utilizes simultaneous principal curves to generate the smooth representation of the lineages of MST, then conducts orthogonal projection of cells onto the principal curves. Finally, the slingshot calculates the arc length from the start point to all the projected points on the principal curve of cells as the pseudotime. However, the pseudotime is very susceptible to errors in generating MST. Monocle3 follows a similar workflow learning a principal graph in the low-dimensional space and calculating by geodesic distance. In practice, it is hard to prevent the construction of an inaccurate MST because both dimension reduction and clustering are challenging unsupervised learning tasks. To address this issue and produce a more robust pseudotime, we infer the pseudotime of the cells first. Therefore, we can use this pseudotime as a weak label to contribute to the modification process of the inferred trajectory.

One basic assumption for the trajectory inference task is that the cells closer to each other on the trajectory have a similar gene expression profile. This assumption is valid for the low-dimensional space generated by a dimension reduction algorithm. Therefore, those cells belonging to the same development stage have similar latent in the low-dimension space. To verify this assumption, we conducted experiments on pseudotime inference using the true cell types instead of clustering results. We chose the euclidean distance as the metric of similarity between cells. First, we selected the start group of cells as the start point and calculate the euclidean distance between the center points of the start group and other groups of cells as its pseudotime. Although the idea and computation were simple, we found that the pseudotime of the cells can be inferred very accurately with the true label.

When applying pseudotime inference by euclidean distance without the true cell type, the accuracy drops significantly because of two aspects. The first is that when replacing the true cell type with clustering results, some cells are grouped into the wrong cluster because of the limited capacity of the clustering method. Secondly, it is a challenging task for the clustering method to infer the correct number of cell types, and an incorrect cluster number will result in poor clustering accuracy and cause the constructed graph to be inaccurate in the following step. Hence, the pseudotime inference accuracy was degraded.

To address these issues, we proposed a robust pseudotime inference algorithm utilizing multiple scDHA clustering results at different resolutions from coarse-scale (5 clusters) to fine-scale (10 clusters). Algorithm 1 illustrates the pseudotime inference algorithm. It requires the clustering result of the data set obtained by scDHA as input. In addition, one or multiple cells at the start point are required input as the prior information to identify the starting cluster. The pseudotime inference algorithm starts with the scDHA clustering result set *k* as 5, the Algorithm 1 first determines that a cluster is the starting cluster based on prior knowledge of the starting cells given by the user, and the mode cluster of the given starting cells is defined as the starting cluster. Algorithm 1 assigns the pseudotime of cells in the starting cluster to 0. In the second step, traverse through the clusters in the clustering result except for the starting cluster, calculate the euclidean distance from the starting cluster center point and assign the pseudotime of cells in the corresponding clusters. Repeat the above two steps for *k* from 5 to 10, Algorithm 1 obtains six pseudotime values for all cells. The last step is to sum the six pseudotime results element-wisely and divide it by six to generate the final pseudotime.

To verify the effect of the choice of the range of k on scTEP’s performance, we tested scTEP with multiple maximum *k* value setting from 11 to 20. The clustering result obtained from a larger maximum *k* value setting discriminated cells at a more fine scale. Hence, there are more differences between the pseudotime of the cells generated by Algorithm 1. In general, a larger maximum *k* value is beneficial, but insignificant, to pseudotime inference accuracy at the cost of running the clustering method a few more times. In the trade-off between accuracy and time efficiency, we set the default range of *k* value from 5 to 10. Users can set the minimum and maximum *k* values based on their needs.
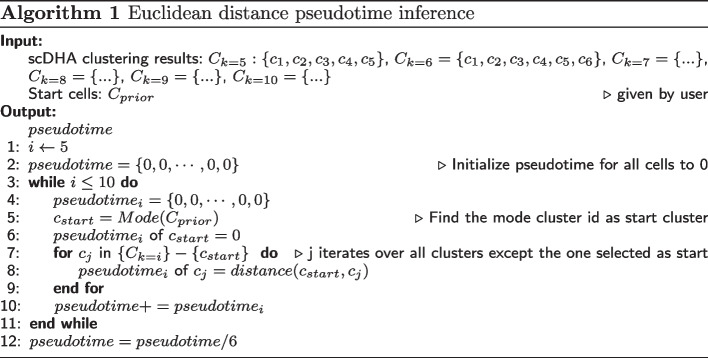


### Trajectory inference

Figure 6d shows the trajectory inference procedure. The last part of the pipeline is to utilize the latent obtained by scDHA to infer the trajectory of the cells. We choose the igraph [39] package to determine the MST from the scDHA latent. The first step is to calculate the center of the clusters, which will be the vertices in the MST representing the center of the cells belonging to that cluster. We calculate a distance matrix of the center of clusters using the euclidean distance. Then we use the distance matrix as the adjacency matrix to build an undirected fully connected graph. Note that the euclidean distance between two vertices is the weight of the edge connecting those vertices on the graph and the average pseudotime is the attribute of vertices. Next, we construct the MST from the undirected fully connected graph using the igraph package. Lastly, we select the mode cluster index in the prior start cells given by the user as the start vertex. Therefore, we obtain a directed tree with the start vertex as the root vertex.

Slingshot and other methods have demonstrated that the MST algorithm has the state-of-the-art capacity to construct a graph for the trajectory inference task. Although these methods calculate the pseudotime differently, one common property is that they no longer modify the MST’s structure. However, we observed that the MST algorithm has poor robustness for the trajectory inference task. One drawback of the MST algorithm is that it is committed to constructing an undirected graph. Therefore, the MST algorithm can generate a tree with the minimum sum of edge weights while having a reversed order of vertices compared to the ground truth development stages. Another drawback is that the MST algorithm depends entirely on the weights between edges and neglects the information on the vertex’s attributes. While the vertex’s attributes are the profile of the cluster of cells, they are very beneficial to the trajectory inference task. When we compared the MST with ground truth, we observed that the order of vertices in the MST does not match the development stages of cells on some data sets. We conclude that the previously mentioned drawbacks are related to this issue. To solve these problems, we proposed a method to fine-tune the MST based on the induced pseudotime from the previous part in the pipeline.

The Pseudotime MST fine-tune algorithm presented in Algorithm 2 requires 3 inputs: (i) a directed MST, (ii) the pseudotime for vertices of the MST, and (iii) the root vertex. The algorithm starts with the root vertex $v_{start}$, finds all the descendent vertices and neighbors of $v_{start}$, represented as $V_{descendents}$ and $V_{neighbors}$ respectively. It then finds the descendants that connect to $v_{start}$ directly by intersecting $V_{descendents}$ and $V_{neighbors}$, represented as $V_{descendents\_direct}$. The essential idea of the pseudotime MST fine-tune algorithm is to modify the MST to make its order of vertices consistent with the pseudotime inferred from the previous part. To achieve this, we find the vertex $v_{min}$ with minimum pseudotime from the descendent vertices $V_{descendents}$. By comparing the pseudotime of the root vertex $v_{start}$ with $v_{min}$, we analyzed if the order of the root vertex with its descendants is correct. If the pseudotime of root vertex $v_{start}$ is greater than $v_{min}$, we swap the position of $v_{start}$ and $v_{min}$, the weight of the edges that connect $v_{start}$ and $v_{min}$ with their neighbor vertices are recalculated. We then traverse the subtrees starting with vertices in $V_{descendents\_direct}$ and conduct the pseudotime MST fine-tune algorithm on the subtrees. After the pseudotime MST fine-tuning algorithm is finished we have a sorted MST $G_s$ in which the lineages are consistent with the pseudotime. 
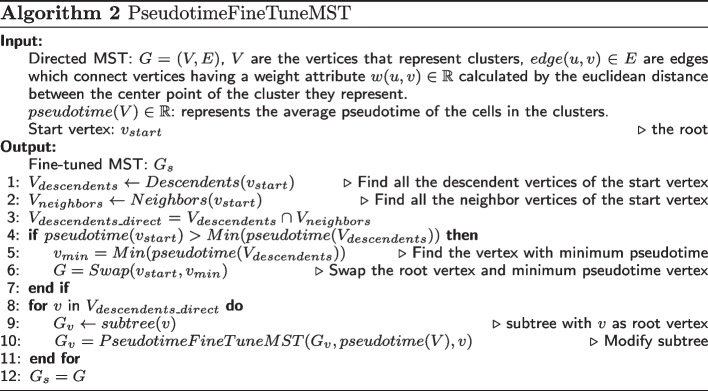


## Conclusions

This paper presented a novel approach toward trajectory inference and pseudotime inference using single-cell RNA sequencing data. We call this approach scTEP. scTEP utilizes the ensemble clustering results to infer robust pseudotime. Utilizing pseudotime, scTEP further fine-tunes the MST to enhance its accuracy and robustness. In addition, scTEP adopts the modularity design idea and consists of several major components in terms of clustering, dimension reduction, pseudotime inference, and trajectory inference. Therefore, it is convenient to incorporate other state-of-the-art methods for the individual components. Experimental results demonstrate the effectiveness of scTEP.

## Data Availability

The gold standard data sets are collected by Saelens et al. in [15]. The gold standard data sets can be downloaded for free at https://zenodo.org/record/1443566#.Y3q1fnbMKUl. The data sets we collected can be downloaded from the NCBI Gene Expression Omnibus by the accession number provided in Table 1.

## Abbreviations

scTEP: Single-cell data Trajectory inference method using Ensemble Pseudotime inference
RNA: Ribonucleic acid
MST: Minimum spanning tree
KNN: K-nearest-neighbor
HIM: Hamming–Ipsen-M-ikhailov
